# Combining two grading systems: the clinical validity and inter-observer variability of the 1973 and 2004 WHO bladder cancer classification systems assessed in a UK cohort with 15 years of prospective follow-up

**DOI:** 10.1007/s00345-020-03180-5

**Published:** 2020-04-07

**Authors:** Paramananthan Mariappan, Paul Fineron, Marie O’Donnell, Ruth M. Gailer, David J. Watson, Gordon Smith, Kenneth M. Grigor

**Affiliations:** 1 Edinburgh Urological Cancer Group, Department of Urology, Western General Hospital, Crewe Road South, Edinburgh, EH4 2XU UK; 2 University of Edinburgh, Edinburgh, UK; 3 Department of Pathology, Western General Hospital, Edinburgh, EH4 2XU UK

**Keywords:** Bladder cancer, Non-muscle invasive bladder cancer (NMIBC), WHO classification, ISUP classification, Prognosis, Long-term follow-up

## Abstract

**Purpose:**

Paucity of reliable long-term data on the prognostic implications of the 2004 WHO bladder cancer classification system necessitates utilisation of both this and the 1973 grading systems. This study evaluated, in noninvasive (pTa) bladder tumours, the prognostic value of the 2004 system independently and in combination with the 1973 system while establishing concordance between tertiary centre uropathologists.

**Methods:**

We used a cohort of non-muscle invasive bladder cancer (NMIBC) patients diagnosed between 1991 and 93 where tumour features were gathered prospectively with detailed cystoscopic follow-up data recorded over 15 years. Initial grading was by one senior expert uropathologist (UP1) using the 1973 WHO classification alone. Subsequently, two other expert uropathologists (UP2 and UP3), blinded to the previous grading, re-evaluated the pathology slides and graded the tumours using both the 1973 and 2004 systems. Association between grade and recurrence/progression was analysed and the Cohen Kappa test assessed concordance between pathologists.

**Results:**

Of 370 new NMIBC, 229 were staged noninvasive (pTa). Recurrence rates were 46.2% and 50.0% for LGPUC (low-grade papillary urothelial carcinoma) and HGPUC (high-grade papillary urothelial carcinoma), respectively, while progression was seen in 3.9% and 10.0% of LGPUC and HGPUC, respectively. Concordance between uropathologists UP2 and UP3 for the 2004 and 1973 systems was good (Kappa = 0.69) and fair (Kappa = 0.25), respectively.

**Conclusions:**

With good inter-observer concordance, the 2004 WHO classification system of noninvasive bladder tumours appears to accurately predict recurrence and progression risks. The combination of both grading systems to low-grade tumours allows further refinement of the natural history.

## Introduction

The management of patients with bladder cancer, in the absence of reliable biomedical markers, is dependant largely on the Histopathological interpretation of cellular appearance. To this end, the WHO introduced the 1973 classification system [1]. To date, this forms the foundation of many clinical trials, nomograms, and risk calculators [2–4]. In non-muscle invasive bladder cancer (NMIBC), which accounts for up to 80% of new bladder cancers, the risk of recurrence can be as high as 80% and the risk of progression to muscle invasive cancer can be up to 20%. The tumour grade forms a useful surrogate of tumour behaviour [3, 5].

Due to the apparent lack of reproducibility and accuracy of the 1973 classification system in part due to poor description of criteria for each grade [6], a new grading system was suggested 25 years later [7]—as this grading system was published in the 2004 edition of the “blue books” WHO classification of tumours, it became popularised as the 2004 classification of urothelial carcinoma grading [8].

There is paucity of reliable long-term data on the prognostic implications of the 2004 classification system and a reluctance of many clinicians to abandon a system that they are familiar with and on which much of the long-term prognostic studies are based. This has resulted in a recommendation to utilise both systems, including by bodies like the EAU [9] and the UK Royal College of Pathologists [10].

Having previously published on the long-term follow-up of Ta tumours using the 1973 classification [11, 12] in tumours during the era of trials that contributed to the EORTC risk tables, we designed this study to: (a) assess the predictive value of the 2004 classification system alone and in combination with the 1973 system in relation to recurrence and progression and (b) to assess concordance between pathologists using both grading systems.

## Patients and methods

We accessed the archived histo-pathology slides for all new bladder tumour patients used in the previous study from our group [12]. Data pertaining to recurrence and progression had already been analysed previously from this cohort of patients with NMIBC. Our pathologists were blinded to the follow-up findings.

Three experienced uropathologists working in a university hospital tertiary centre (with over 10 years experience each as specialist uropathologists) were involved in this study and evaluation of tumour histo-pathology. Uropathologist (UP1) who had reviewed the histo-pathology slides in the 1990s reviewed the slides once more and defined tumour grades using only the WHO 1973 classification system. The other two uropathologists (UP2 and UP3) were asked to independently classify tumours using the 1973 and the 2004 classification systems to define pathological grade. The uropathologists involved in this study were not permitted to compare their grading results.

Tumour stage was defined using the TNM classification. Only tumours which had been accepted as being pTa by all three uropathologists were included in the analysis.

Using the 2004 classification system, for consistency in description, low- and high-grade Ta (noninvasive) tumours were called low-grade papillary urothelial carcinoma (LGPUC) and high-grade papillary urothelial carcinoma (HGPUC), respectively. Other descriptions were papilloma and papillary urothelial neoplasia of low malignant potential (PUNLMP).

Only patients with new tumours diagnosed between 1991 and 1993 were included in the study. The database available included only patients with urothelial carcinoma. Exclusion criteria for analysis were recurrent tumours, pTx, pT1, and pT2 tumours. The follow-up regime and intravesical chemotherapy employed were based on the guidelines of the time and all patients were discussed at a multi-disciplinary meeting, as published previously [12]. Follow-up flexible cystoscopy commenced at 3 months after primary treatment which was either by transurethral resection of bladder tumour (TURBT) or biopsy and diathermy (B&D). This was followed by a flexible cystoscopy at 6-, 9-, and 12-month cystoscopy prior to annual follow-up for those remaining tumours free.

Recurrence was defined as a pathologically proven tumour. Progression was defined as a recurrence with a higher grade to what was noted at the initial diagnosis and/or development of pT1 or pT2 disease. The pathology of the recurrent tumour was defined by UP1 who acted as the reference pathologist.

Data were analysed using the SPSS ver 16.0 software. Recurrence and progression-free survival were evaluated using Kaplan–Meier curves and the log-rank test. Concordance between uropathologists was evaluated using the Cohen Kappa (*κ*). Accepted *κ* values for fair agreement (0.21–0.40), moderate agreement (0.41–0.60), and good agreement (0.61–0.8) were used [13].

## Results

From a total of 508 patients with urothelial carcinoma, 370 (72.8%) consecutive NMIBC patients were assessed, of whom there were 229 (61.9%) patients with pTa tumours—89 (38.9%), 109 (47.6%), and 31 (13.5%) were defined as Grades 1, 2, and 3, respectively, by UP1 using the 1973 classification system. Of these patients, 47 (23.7%) with G1 and G2 tumours were excluded from the analysis due to the finding of some possible lamina propria invasion on review by any one of the three uropathologists. In the total NMIBC cohort, 80 (21.6%), 61 (16.5%) were categorised pT1 and pTx, respectively, by UP1, and, therefore, excluded from evaluation by the other two uropathologists.

Of the patients with pTa cancer included in the analysis (*n* = 182), there were 115 male (63.2%) and 67 female (36.8%) patients with a mean age of 69.7 years (range: 48.6–91.1) at the time of diagnosis. The median duration of surveillance was 92 months (3–174 months).

All 31 patients with Grade 3 pTa tumours were classed as high grade using the 2004 grading system by both UP1 and UP2. Eight (25.8%) of these patients had cis in the initial histology. Of the Grade 3 pTa tumours, 21 (67.7%) were small (< 3 cm) and 18 (58.1%) were single at presentation.

Of the 151 (Grade 1 or Grade 2) pTa patients, single and multiple tumours were found in 134 (88.7%) and 17 (11.3%) patients, respectively. Small tumours (< 3 cm) and large tumours (≥ 3 cm) were found in 106 (70.2%) and 45 (29.8%). A single post-TURBT instillation of mitomycin C was given following the initial TURBT in 11 (13.3%), 12 (17.7%), and 3 (9.7%) G1pTa, G2pTa, and G3Ta patients, respectively; while a 6-week course of weekly mitomycin C was used in 3 (3.6%) G1Ta and 4 (5.9%) G2Ta patients. Intravesical BCG was used in 8 (25.8%) of the G3Ta patients and upon progression in 4 (5.9%) G2Ta and 1 (1.2%) G1Ta patients.

Both Grade 1 and Grade 2 patients had a larger proportion of LGPUCs (Table 1). A small number of Grade 1 neoplasm cases were reclassified as PUNLMP using the WHO 2004 classification system. HGPUC were only seen in a small proportion of those labelled as Grade 2 using the older classification system—8/68 (11.8%) and 11/68 (16.2%) patients who were initially classified as Grade 2 (1973 classification system) were labelled high grade by UP2 and UP3, respectively, using the later classification system.

**Table 1:** WHO 1973 and 2004 grading for tumours graded as G1, G2, and G3 by UP1

UP1 WHO 1973	UP2 WHO 1973	UP2 WHO 2004	UP3 WHO 1973	UP3 WHO 2004
G1 = 83	G1 = 58	Papilloma = 0	G1 = 39	Papilloma = 0
	G2 = 25	PUNLMP = 6	G2 = 44	PUNLMP = 2
	G3 = 0	LGPUC = 74	G3 = 0	LGPUC = 81
		HGPUC = 0		HGPUC = 0
G2 = 68	G1 = 21	Papilloma = 0	G1 = 6	Papilloma = 0
	G2 = 45	PUNLMP = 0	G2 = 62	PUNLMP = 0
	G3 = 2	LGPUC = 60	G3 = 0	LGPUC = 57
		HGPUC = 8		HGPUC = 11
G3 = 31	G3 = 31	HGPUC = 31	G3 = 31	HGPUC = 31

Using grade alone as predictor of recurrence, the 2004 system revealed a trend of rising recurrence from PUNLMP through to HGPUC, a trend that the older classification system failed to demonstrate (Table 2).

**Table 2:** Recurrence rate (risk of developing at least one recurrence through out the duration of follow-up)

Tumour grade	Recurrence (%)
1973 Grade (grading by UP1)	
G1	50/83 (60.2)
G2	34/68 (50.0)
2004 Grade (where both UP2 and UP3 concurred on the grade)	
PUNLMP	3/6 (50.0)
LGPUC	41/85 (48.2)
HGPUC	4/6 (66.7)

**Fig. 1:**
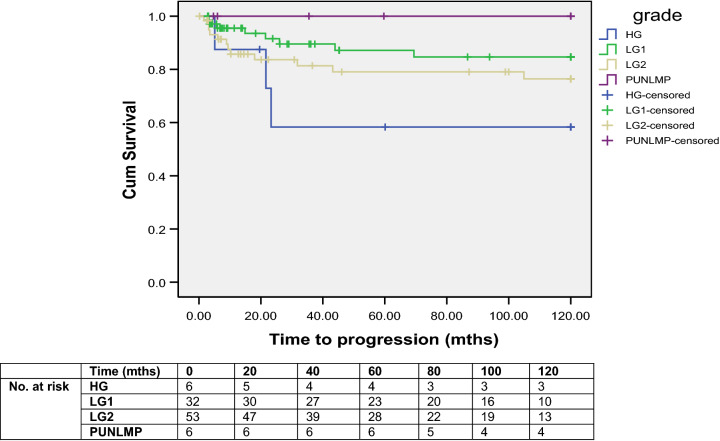
Progression-free survival in patients initially classified as G1 or G2 by UP1 (*HG* High-Grade, *LG1* Low-Grade-Grade 1, *LG2* Low-Grade-Grade 2, *PUNLMP* papillary urotheliam neoplasia of low malignant potential)

The risk of progression was proportionate to the tumour grade as determined by reclassification using the 2004 system—the addition of the 1973 classification system to the 2004 system (i.e., categorising as low-grade G1 and low-grade G2) allowed further sub-stratification of the low-grade tumours with a clear separation in risk of progression between the two sub-strata (Fig. 1). Inter-observer concordance/agreement was deemed to be ‘good’ when using the two-tier 2004 classification system and, at best, ‘moderate’ with the three-tier 1973 classification system (Table 3).

**Table 3:** Concordance/agreement between uropathologists

Pathologists	Kappa (*κ*), *p* value, agreement
1973 WHO classification system	
UP1 vs UP2	0.417, < 0.001, moderate
UP1 vs UP3	0.330, < 0.001, fair
UP2 vs UP3	0.253, 0.001, fair
2004 WHO classification system	
UP2 vs UP3	0.693, < 0.001, good

## Discussion

The WHO 1973 standard for classification and grading of bladder tumours is often considered a robust clinically proven, widely used, time-tested, and reasonably reproducible method for pathologic reporting, and is recommended with minor modifications—thus despite the introduction of the 2004 system, the EAU and UK Royal College of Pathologist guidelines (amongst others) recommend the use of both classification systems mainly to obtain long-term results—at least until long-term validation data of the 2004 classification system are available [14]. The newer classification system has been around for more than a decade and publications evaluating its longer term validity are somewhat scarce, to our knowledge, but nonetheless deemed reliable [9]. The 2004 classification system is still being recommended to be used alongside the 1973 system as best practice, despite a recent (2016) update of the classification system [15] and some recent evidence in favour of the 1973 classification system in pT1 cancers [16]. It must be noted that the 2004 system has not been used in any prognostic risk-models to date [9].

The combination of the two classification systems and its benefits is one of the aspects which we have explored in this study—whilst, to our knowledge, this is the first prospectively maintained cohort of patients with long-term follow-up being used to evaluate the new classification system, we also provide a unique demonstration of the value in combining the two systems. The low-grade G1 and low-grade G2 categories revealed some separation in the Kaplan–Meier curves. Despite a non-statistically significant difference (possibly consequent to a modest sample size), this combination offers an enhanced prognostic value—we, therefore, feel that this additional prognostic information, in the absence of reliable bio-markers, could help to better inform patients and select those suitable for adjuvant chemo or immunotherapy. The WHO 2004 classification system is not directly interchangeable with the WHO 1973 system. We observed a minority of cases previously termed Grade 1 tumours that may represent PUNLMP, but, diagnostically, these are difficult to separate out with confidence. The remaining Grade 1 tumours would be classified as LGPUC using the new system [17]. While the majority of Grade 2 tumours would be classified as LGPUC on review, there is a significant minority of Grade 2 tumours that would be re-graded as HGPUC using the new system. Almost all noninvasive Grade 3 neoplasms will be allocated to the HGPUC group using the new system [17].

### Prediction of recurrence and progression

The recommendation by the Royal College of Pathologists is that PUNLMP is not used by UK pathologists as consistency in discriminating this entity from an LGPUC or Grade 1pTa Urothelial carcinoma is regarded as exceptionally difficult [10]. It is quite possible that very experienced uropathologists might be better at separating these, but this skill is not ubiquitous. In addition, it can also be confusing regarding management as the long-term natural history of PUNLMP is un-known and essentially most clinicians will follow-up as G1pTa. From our study, our expert uropathologists did not categorise many low grade (from the 1973 system) into PUNLMP and in the ones, that were given this label, the risk of recurrence appeared not dissimilar to the LGPUC category. The observation of a lower proportion of PUNLMP was equally reported by others [18]. More importantly, the risk of progression was indeed negligible in the PUNLMP category. This appeared to be in-keeping with findings from Holmang et al. [19, 20], who, on evaluating a cohort of 95 patients with PUNLMP, found recurrences in 35% and no progressions over 5 years of follow-up (supporting the notion of difficulty in making this diagnosis and separating from LGPUC). Conversely, patients with LGPUC had recurrence and progression rates of 71% and 4%, respectively. Similar findings were reported by others who also evaluated the relationship between LGPUC and some molecular markers [21–23]. However, there are varying reports from other authors, with progression observed even in PUNLMP (range 0–8%) [24]. In our opinion, this heterogeneity of recurrence rates (and even progression) is not necessarily the result of heterogeneity in tumour behaviour, rather more likely the effect of differing surgical quality [25]. The improved quality of initial tumour resections has now produced a significant reduction in recurrence rates and, consequently, will make comparisons between contemporary and historical series difficult, if not untenable.

Using grade alone as a predictor of recurrence, the 2004 system revealed a trend of rising recurrence from PUNLMP through to HGPUC, a trend that the older classification system failed to demonstrate (Table 3). Risk tables using the 1973 classification system [3] had consistently showed that the grade was a better predictor of progression compared to recurrence, which is better determined by multiplicity and size of the tumour. Equally, the 2004 system appeared to be more representative of the tumour natural history in relation to progression. Cao and colleagues [26] demonstrated that the 2004 classification system predicted recurrence and progression better than the 1973 system, particularly in noninvasive tumours, while Otto et al. [27] and Pelluchi et al. [16] revealed that the 1973 classification system was better in pT1 cancers regarding the prediction of progression. In addition, Burger et al. revealed that the 2004 system was more accurate in predicting recurrence in patients with regular onset bladder cancer compared to those with the early onset bladder cancer (> 45 years of age) [28].

### Inter-observer variability

We ensured that the three pathologists involved in the study did not confer on the grading ascribed to a tumour. All G3 tumours became HGPUC with the 2004 classification system, i.e., there was no doubt between all three uropathologists regarding the categorisation. Our uropathologists appeared to have more concordance when using the 2004 system compared with the 1973 one, i.e., there appears to be lesser inter-observer variability with th“e 2004 system. It is well accepted within Pathological literature that a two-tier grading system is easier to use and provides less inter-observer variability than a three-tier system where there is a tendency to “drift to place cases in the middle group”. The recent EAU guidelines conclude, “The published comparisons have not clearly confirmed that the WHO 2004 classification system provided better reproducibility than the 1973 system” [9]. In Germany, May and colleagues [18], from a retrospective review of 200 patients, confirmed a better concordance between uropathologists when using the 2004 classification system as compared to the 1973 one—Kappa (*κ*) value was up to 0.52 in a comparison between two pathologists probably for similar reasons to ours. An analysis of a small Australian patient cohort also revealed a similar inter-observer variability as our recent analysis [29]. Mangrud and colleagues [30], analysing 193 bladder tumours with a median follow-up of 75 months, revealed that a Kappa value of 0.68 for inter-observer agreement in Grade 1 and 2 tumours (using the 1973 classification system) and inter-observer agreement for the 2004 system was 0.7, concluding that neither system was prognostically superior than the other. To negate this inter-observer variability and the potential change in clinical management [31], certainly the Urology community and much of the practice in UK cancer centres, like ours, rely on consensus of opinion of at least two expert pathologists in most bladder cancer evaluations.

High throughput with next-generation sequencing has now allowed us to better understand the genomic patterns in bladder cancer [32] and potentially allow for targeted personalised treatment̄—however, there is an observed heterogeneity in the association between molecular profiles and clinical behaviour [33]. Burger et al. performed a prospective evaluation of the association between recurrence and progression (over 3 years) against the 1973 classification system, 2004 system, and FGFR mutation status in patients with NMIBC. The authors concluded that both grading systems provided valuable prognostic information, particularly in the ability to predict progression. FGFR mutation appeared to act as an adjunct to the prognostication in only High-Grade cancer [34]. Intuitively, reliable biomolecular markers (when available) are likely to not only improve prognostication in the future, but also provide a more uniform platform for consistent reporting and, therefore, molecular pathology and bio-markers should be the way forward in introducing objectivity, consistency, and reproducibility to prognostication in NMIBC [33]. In the future, with the reduced cost and potential universally accessible genomic analysis using next-generation sequencing, molecular evaluation should be an adjunct to (and not a replacement of) histo-pathological assessment [35], especially as some early data already allude to, for example, proliferative differences based on molecular markers [23].

Although the number of patients in our study appears to be at par, if not larger, and the follow-up appears to be longer than several other publications, the cohort is still quite modest. It would appear that most other publications also referred to the sample size as tumour number as opposed to number of patients [18, 30]. The modest size of our cohort also precluded the simultaneous evaluation of recurrence/progression risk by stratifying to tumour features (number and size)—the emphasis of this work being the value of the WHO grading systems, not dissimilar to the approach of other authors [18, 28]. Combination of raw data from the other groups may help in achieving a much larger cohort to validate our observations. T1 tumours were not included as the outcomes from the 1990s are unlikely to mirror the current approach, especially in light of the more ubiquitous recent practice of early re-TURBT in this group of patients—a practice that was not routine in earlier series. Grade 3 tumours would have similar implications. While we did have information about presence of cis in the initial histology, other prognostically relevant features in current pathology reports (such as lympho-vascular invasion and micropapillary appearance), were not discernible for this cohort. Long-term outcomes from this cohort may also not necessarily represent findings in contemporary practice, where there is improved quality of the initial surgery, increased usage of immediate post-operative chemotherapy, and adjuvant chemo/immunotherapy. However, this cohort was chosen for its proximity to those in the era of the trials that were used in the development of the EORTC risk tables; the use of post-operative instillation of chemotherapy was also part of the MRC trial [2]; and we had reliable prospective long-term follow-up. The difference in progression risk between LG-G1 and LG-G2 noted in this study is unlikely to have been altered even if there was a hypothetical wider use of a single post-operative chemotherapy instillation as the further meta-analysis of previous clinical trial data demonstrated that the tumour grade did not affect response to the immediate post-TURBT intravesical chemotherapy [36].

## Conclusion

From this cohort of NMIBC with prospective long-term follow-up, the 2004 WHO classification system appears to better predict the risk of progression and has lower inter-observer variability. Augmenting this grading system with the 1973 WHO classification system appears to improve prognostic value. We recommend further assessment with a larger multi-centre cohort of contemporary patients with long-term follow-up.
